# Early Breakfast

**Published:** 1860-05

**Authors:** 


### EARLY BREAKFAST.

BREAKFAST should be eaten in the morning, before leaving the house for exercise, or labor of any description; those who do it will be able to perform more work and with greater comfort and alacrity, than those who work an hour or two before breakfast. Besides this, the average duration of the life of those who take breakfast before exercise or work, will be a number of years greater than of those who do otherwise. Most persons begin to feel weak after having been engaged five or six hours in their ordinary avocations; a good meal, reïnvigorates, but from the last meal of the day until next morning, there is an interval of some twelve hours; hence the body in a sense is weak, the stomach is weak, and in proportion can not resist deleterious agencies, whether of the fierce cold of mid-winter, or of the poisonous miasm which rests upon the surface of the earth, wherever the sun shines on a blade of vegetation or a heap of offal. This miasm is more solid, more concentrated, and hence more malignant, about sunrise and sunset, than at any other hour of the twenty-four, because the cold of the night condenses it, and it is on the first few inches above the soil in its most solid form; but as the sun rises, it warms and expands and ascends to a point high enough to be breathed, and being taken into the lungs with the air, and swallowed with the saliva into the stomach, all weak and empty as it is, it is greedily drank in, thrown immediately into the circulation of the blood, and carried directly to every part of the body, depositing its poisonous influences at the very fountain-head of life. When in Cuba many years ago, we observed that the favorite time for travel was midnight; and the older merchants of Charleston may remember that when deadly fevers prevailed in hot weather, they dared not ride into town in the cool of the morning or evening, but midday was accounted the safest. We know, from many years' living in New-Orleans, that it was when the evenings and mornings were unusually cool, balmy and delightful, the citizens prepared themselves for still greater ravages of the deadly epidemic for the first few days following.

If early breakfast was taken, in regions where chill and fever, and fever and ague prevail, and if in addition, a brisk fire were kindled in the family-room, for the hour including sunset and sunrise, these troublesome maladies would diminish in any one year, not ten-fold, but a thousand-fold, because the heat of the fire would rarefy the miasmatic air instantly, and send it above the breathing-point. But it is "troublesome" to be building fires night and morning all summer, and not one in a thousand who reads this will put the suggestion into practice, it being no "trouble," requiring no effort, to shiver and shake by the hour, daily, for weeks and months together; such is the stupidity of the animal, man!

